# Juvenile stroke during long-term therapy with adalimumab

**DOI:** 10.1016/j.clinsp.2026.100873

**Published:** 2026-02-19

**Authors:** Ana C. Fiorini, Fulvio A. Scorza, Josef Finsterer, Carla A. Scorza

**Affiliations:** a Department of Speech Therapy, Escola Paulista de Medicina, Universidade Federal de São Paulo (EPM/UNIFESP), São Paulo, SP, Brazil; b Postgraduate Studies Program in Speech Therapy, Pontifícia Universidade Católica de São Paulo (PUC-SP), São Paulo, SP, Brazil; c Escola Paulista de Medicina, Universidade Federal de São Paulo (UNIFESP/EPM), São Paulo, SP, Brazil; d Neurology Department, Neurology & Neurophysiology Center, Vienna, Austria

**Keywords:** Crohn's disease, Adalimumab, Ischemic stroke, Immunology, Juvenile stroke

## Correspondence

Long-term treatment of Crohn's disease with adalimumab is generally highly effective and is tolerated without major side effects.^1^ In individual cases, however, adalimumab can lead to side effects, as in the following patient.

The patient is a 29-year-old man who suffered a right-sided ischemic thalamic stroke at the age of 26. The stroke was classified as of undetermined cause after a comprehensive investigation of the underlying cause was inconclusive. Clinically, the stroke presented with diffuse headache up to a Visual Analogue Scale (VAS) of 5, neck stiffness, disorientation, and memory impairment. Blood tests, including inflammatory markers, were non-informative except for mild hyperlipidemia. Cerebral MRI showed a subacute ischemic lesion in the right thalamus. Magnetic Resonance Angiography (MRA), carotid ultrasound, Transthoracic Echocardiography (TTE), Transesophageal Echocardiography (TEE), 24-hour ECG and 24-hour blood pressure measurement as well as blood tests for coagulopathy, including lupus anticoagulans, antiphospholipid antibodies, protein-S, protein-C, homocysteine, antithrombin III, connective tissue disease and diabetes, were unremarkable. The medical history was negative for chronic stress or substance abuse. A patent foramen was ruled out on TEE. The patient refused the implantation of a loop recorder. Acetylsalicylic acid was administered in addition to rosuvastatin. After a four-month rehabilitation phase, the initial deficits were completely resolved. His medical history revealed Crohn's disease, which was diagnosed at the age of 21. From the age of 22, he received adalimumab (40 mg every two weeks), which he still takes today. In his family history, his maternal grandmother, who was a smoker, was diagnosed with an ischemic stroke. His psychosocial situation was stable at the time of the stroke.

He was readmitted at the age of 29 due to evening fatigue. He complained of severe work stress, which led to chronic overload with vegetative symptoms and depressive moods as well as sleep deficits, after recently accepting a new job. A renewed investigation into the causes of the previous stroke revealed that he had developed temporary hypertension after the stroke and was receiving metoprolol (25 mg/d), which was discontinued due to repeated hypotension. Further investigations into the causes of the previous stroke, including cerebral MRI and MRA of the intra- and extracranial arteries, were inconclusive. A telemetry recording for several days did not reveal Atrial Fibrillation (AF) or any other severe arrhythmias. Hyperlipidemia was well controlled. The patient tested negative for the Leyden mutation and the c.20210G>*A* prothrombin mutation. TTE, TEE, 24-hour ECG, 24-hour blood pressure measurement, cardiac MRI, carotid ultrasound, and blood tests were unremarkable. The cerebral MRI still showed the old right-sided thalamic stroke. The evening fatigue disappeared after he left his current job. The only risk factor, apart from mild hyperlipidemia, was adalimumab.

The presented patient is of interest because of an ischemic stroke of undetermined cause in the right thalamus at the age of 26-years. Apart from mild hyperlipidemia, which was well controlled with rosuvastatin, no cause for the juvenile stroke could be found by repeated, extensive examinations. Hyperlipidemia was ruled out as a cause because no microangiopathy could be detected, the hyperlipidemia was mild and well controlled with a statin, and no other sites of micro- or macroangiopathy could be found.

At the age of 29, it was first suggested that adalimumab may have played a causative role in the development of the previous stroke. The assumption that adalimumab could be a possible etiological factor is supported by a previous report about adalimumab as the possible cause of juvenile ischemic stroke^2^ and by its side effect profile, suggesting an increased risk of stroke.^3^ This includes Heart Failure (HF) or impaired systolic function as a cardiovascular risk factor that may be involved in the development of a stroke. HF can be complicated by arrhythmias, including AF. Both AF and HF are risk factors for stroke. Since the cardiac examination was performed days after the stroke, it cannot be ruled out that transient arrhythmias or HF were indeed the causes of the cerebral event. There are also indications that TNF-alpha blockers increase the risk of thrombosis.^4^^,^^5^ In a study of 1382,627 patients having experienced adverse reactions to TNF-alpha blockers, 9714 were attributed to thrombosis.^4^ Another pathophysiological explanation for how adalimumab might have caused the stroke is vasculitis.^6^^,^^7^ In a study of 8 patients receiving TNF-alpha blockers, 5 developed cutaneous vasculitis, four peripheral nerve vasculitis, and one renal vasculitis.^6^ However, there are also reports showing that biomarkers of cardiovascular risk decrease with adalimumab.

Alternative causes for the stroke could include atrial fibrillation, transient artery dissection, inflammation due to the underlying inflammatory disease, or a JAK21 mutation. A JAK2 mutation was largely ruled out because the patient showed no signs of myeloproliferative disorders, polycythemia vera, essential thrombocythemia, or primary myelofibrosis, and the family history revealed no genetic cause for the stroke. Although the patient declined loop recording, paroxysmal atrial fibrillation is unlikely as a cause, since no further vascular events, palpitations, or syncope occurred during the three-year follow-up period after the stroke. Crohn's disease cannot be ruled out as a cause of the stroke, but it is also unlikely because the disease was inactive and well-controlled at the time of the stroke. Cerebral vasculitis was excluded based on normal inflammatory markers and a normal MRA. To definitively rule out cerebral vasculitis, black-blood sequences, digital subtraction angiography, or a brain biopsy would have been necessary. A transient dissection could largely be ruled out, as it would have been visible on MRA with a residual intramural hematoma. Limitations of the study include the fact that JAK2 mutations, paroxysmal atrial fibrillation, cerebral vasculitis, and not all hereditary coagulopathies could be ruled out as the cause of the stroke.

In summary, this case suggests that adalimumab may, in isolated cases, be involved in the pathophysiology of juvenile ischemic stroke. Further studies are needed to assess whether adalimumab actually increases the risk of ischemic stroke. Until then, the question of a causal relationship between adalimumab and stroke remains unresolved.

## Analytic method

Case report.

## Data availability statement

Data that support the findings of the study are available from the corresponding author.

## Statement of ethics

The study was approved by the institutional review board. Written informed consent was obtained from the patient for publication of the details of his medical case.

## Compliance with ethics guidelines

This article is based on previously conducted studies and does not contain any new studies with human participants or animals performed by any of the authors.

## Authors’ contributions

Josef Finsterer: Design, literature search, discussion, first draft, critical comments, final approval. Ana C. Fiorini, Fulvio A. Scorza, Carla A. Scorza: Literature search, discussion, critical comments, final approval.

## Funding

No funding was received.

## Declaration of competing interest

The author declares that the research was conducted in the absence of any commercial or financial relationships that could be construed as a potential conflict of interest.
